# Tristetraprolin inhibits gastric cancer progression through suppression of IL-33

**DOI:** 10.1038/srep24505

**Published:** 2016-04-14

**Authors:** Kaiyuan Deng, Hao Wang, Ting Shan, Yigang Chen, Hong Zhou, Qin Zhao, Jiazeng Xia

**Affiliations:** 1 Department of General Surgery and Translational Medicine Center, Nanjing Medical University Affiliated Wuxi Second Hospital, Wuxi 214002, China

## Abstract

Tristetraprolin (TTP) is an adenine/uridine (AU)-rich element (ARE)-binding protein that can induce degradation of mRNAs. In this study, we report that TTP suppresses the expression of interleukin-33 (IL-33), a tumor-promoting inflammatory cytokine, and thereby inhibits the progression of gastric cancer (GC). Overexpression of TTP decreased the level of IL-33, whereas knockdown of TTP increased IL-33 levels. We also discovered that TTP inhibited the proliferation, migration, and invasion of GC cell lines through regulation of IL-33. Furthermore, TTP RNA and protein levels were remarkably reduced in GC and inversely correlated with IL-33 level, and they were also closely associated with depth of invasion, lymph node metastasis, advanced TNM stage, as well as survival rate. Taken together, these findings identified TTP as a downregulator of IL-33, and further suggest that TTP can serve as a novel biomarker for the diagnosis of GC and as a potential therapeutic target for GC treatment.

Gastric cancer (GC) is one of the most common cancers and the second leading cause of cancer-related death globally, with an estimated 951,600 new cases and 723,100 deaths occurring in 2012^1^. Although the incidence and mortality rates of GC have stably declined in the past few years, the prognosis for patients with advanced stages of GC remains poor, and effective therapeutic approaches are still limited^1^,^2^. Hence, it is urgent to identify novel biomarkers for the early diagnosis and treatment of GC.

Immune dysfunction and inflammation are widely recognized to be involved in promoting the initiation and progression of cancer^3^. Pro-inflammatory cytokines, such as interleukins, have been reported to be up-regulated and correlated with worse prognosis in GC^4^. Interleukin-33 (IL-33), the 11th member of the IL-1 family, has also recently emerged as a factor in oncogenesis. IL-33 is first produced as a 30-kDa precursor protein, which is then cleaved by caspase-1 to generate a mature 18-kDa form for secretion. As a ligand for the orphan receptor ST2 (IL-1RL1), IL-33 binds to a receptor complex consisting of ST2 and IL-1R accessory protein. The IL-33/ST2 axis plays a significant role in regulating multiple biological and pathological processes by activating various signaling proteins, including nuclear factor-κB (NF-κB), inhibitor of NF-κB-α, extracellular signal-regulated kinase 1/2 (ERK1/2), mitogen-activated protein kinases (MAPK), and cJun N-terminal kinase-1 (JNK1)^5^,^6^,^7^. Enhanced expression of IL-33 has been found in different cancers, such as breast cancer, non-small cell lung cancer, and hepatocellular carcinoma, and is closely associated with tumor growth and metastasis^8^,^9^,^10^. Additionally, elevated levels of IL-33 have also been discovered in both sera and tumor tissues of GC patients, facilitating GC progression by inducing cell invasion and migration^11^,^12^.

The mRNAs of many pro-inflammatory cytokines are found to be unstable^13^. The stability of these mRNAs is mainly determined by the AU-rich elements (AREs) within their 3′untranslated regions (3′UTR), which may induce mRNA destabilization and degradation through binding to specific proteins^14^,^15^. Tristetraprolin (TTP) is a well-known ARE-binding protein that is involved in post-transcriptional regulation of many pro-inflammatory cytokines and transcription factors, such as IL-23, IL-6, HIF-1α and E2F1. Moreover, TTP also acts as a tumor suppressor by inducing the inhibition of its target oncogenes, and it is often down-regulated in various cancers^16^,^17^,^18^,^19^,^20^. However, the role of TTP in GC remains poorly understood.

In this study, we provide the demonstration that expression of TTP is decreased, and inversely correlated with IL-33 expression, in GC. Additionally, we provide evidence that TTP negatively regulates IL-33 expression, thus further regulating the proliferation, migration, and invasion of GC cell lines. We also discovered that overexpression of TTP suppresses tumor growth in nude mice, and using immunohistochemical analyses, we determined that the level of TTP expression is related to clinicopathological features and to overall survival of GC patients, suggesting that TTP levels could serve as an independent prognostic factor. Our study reveals a tumor-suppressive role for TTP in GC, and indicates the potential application of TTP for treating IL-33-mediated tumor promotion.

## Results

### TTP expression is decreased and is inversely correlated with IL-33 expression in GC

**Figure 1:**
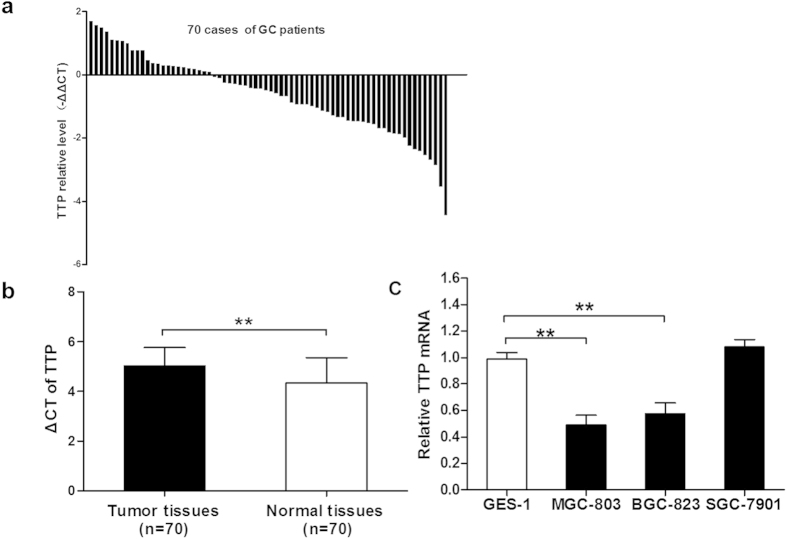
The expression of TTP in gastric cancer tissues and cell lines. (**a**) Relative expression of TTP mRNA in gastric cancer tissues and their corresponding normal tissues was determined by qRT-PCR and expressed as –ΔΔCT. Down-regulation of TTP was found in 65.7% (46/70) of the selected patients. (**b**) Results of qRT-PCR showed that the level of TTP mRNA in tumor tissues was significantly lower than levels in adjacent normal tissues. (**c**) Relative expression of TTP between three gastric cancer cell lines (MGC-803, BGC-823 and SGC-7901) and a normal gastric epithelial cell line GES-1. All data are represented as the mean ± SD of three independent experiments. **P < 0.01.

To understand the role of TTP in the initiation and progression of GC, we first examined the mRNA levels of TTP in 70 paired GC tissues and adjacent normal tissues from Group B by quantitative real-time PCR (qRT-PCR). Decreased levels of TTP mRNA were observed in 65.7% (46/70) cases of tumor tissues in comparison with their matched non-tumorous tissues (Fig. 1a). Meanwhile, the results also showed that the mean TTP mRNA level in tumor tissues was significantly lower than that in adjacent normal tissues (*P* < 0.001) (Fig. 1b). TTP mRNA levels in different cell lines were also determined by qRT-PCR, and the levels of TTP mRNA were lower in MGC-803 (*P* = 0.001) and BGC-823 (*P* = 0.002) cell lines, compared with those in an immortalized human gastric epithelial cell line (GES-1). The mRNA level of TTP was relatively higher in SGC-7901 than that in GES-1, but the difference was not significant (*P* = 0.100) (Fig. 1c).

**Figure 2:**
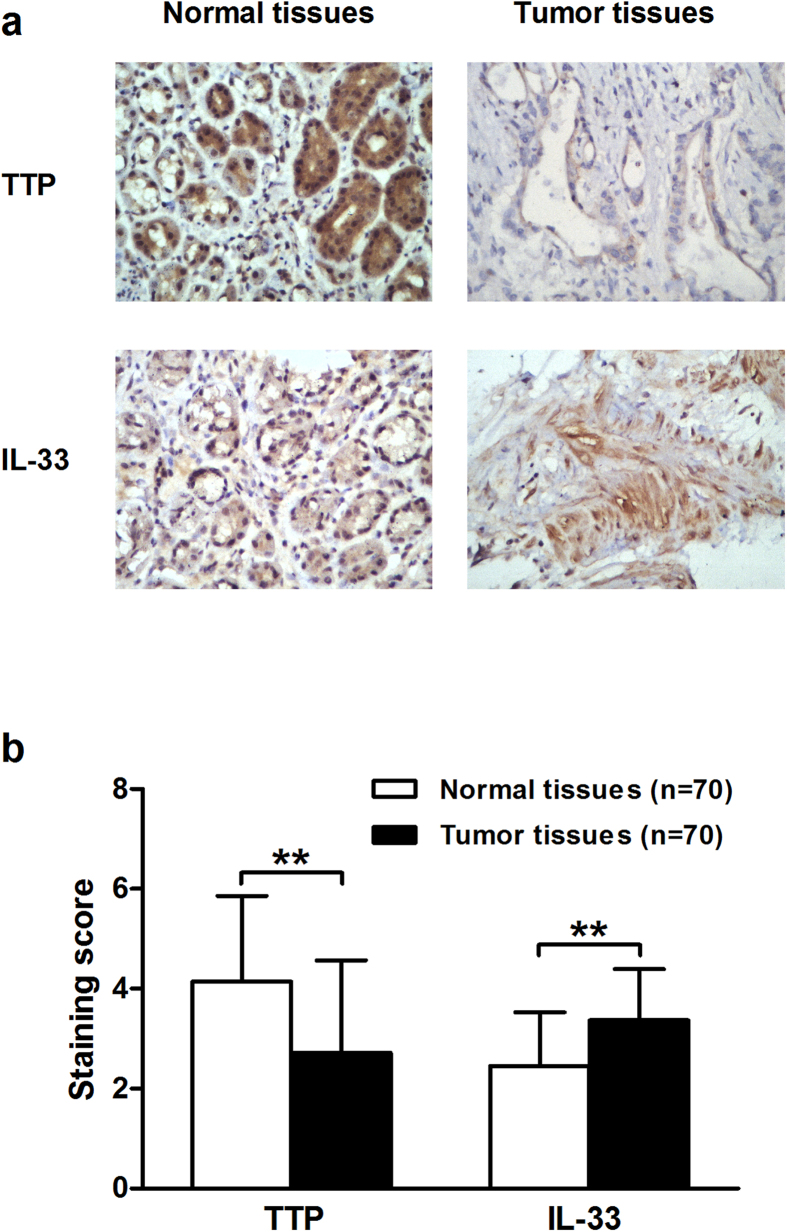
TTP expression is decreased and inversely correlated with IL-33 expression in gastric cancer (Group B). (**a**) Representative immunohistochemical staining of TTP (IHS: top left, 6; top right, 1) and IL-33 (IHS: bottom left, 1; bottom right, 4) in gastric cancer tissues and adjacent normal tissues (400× magnification). (**b**) The normal tissues showed strong positive staining of TTP, while the tumor tissues showed relatively stronger staining of IL-33. All data are represented as the mean ± SD. **P < 0.01.

Similar results were obtained from the assessment of TTP protein expression. By performing immunohistochemical staining in 70 paired tumor tissues and corresponding normal (non-tumorous) counterparts from Group B, we found that TTP staining was dramatically decreased in cancerous tissues (mean immunohistochemistry score (IHS), 2.71 ± 1.86; IHS range, 1–6) compared to adjacent normal tissues (mean IHS, 4.14 ± 1.71; IHS range, 2–6) (*P* < 0.001) (Fig. 2a,b). Collectively, these data suggested that expression of TTP was aberrantly reduced in GC at both the RNA and protein levels.

Next, we analyzed the expression of IL-33 in 70 paired GC tissues and adjacent normal tissues from Group B by immunohistochemistry to investigate the relationship between TTP and IL-33 in GC. The results showed that IL-33 expression was relatively higher in cancerous tissues (mean IHS, 3.37 ± 1.02; IHS range, 1–4) than in normal tissues (mean IHS, 2.46 ± 1.07; IHS range, 1–4) (*P* < 0.001) (Fig. 2a,b), indicating that IL-33 expression was up-regulated in GC and was associated with gastric tumorigenesis.

By using the median IHS value of 3 as a cut-off value, 70 tumor tissues from Group B were divided into TTP-positive (n = 26, IHS > 3) subgroup, TTP-negative (n = 44, IHS ≤ 3) subgroup, IL-33-positive (n = 50, IHS > 3) subgroup, IL-33-negative (n = 20, IHS ≤ 3) subgroup. The result of Pearson’s χ^2^ test demonstrated that TTP expression was inversely correlated with IL-33 expression in GC tissues (Table 1).

### TTP regulates the expression of IL-33 and the activation of ERK1/2

**Figure 3:**
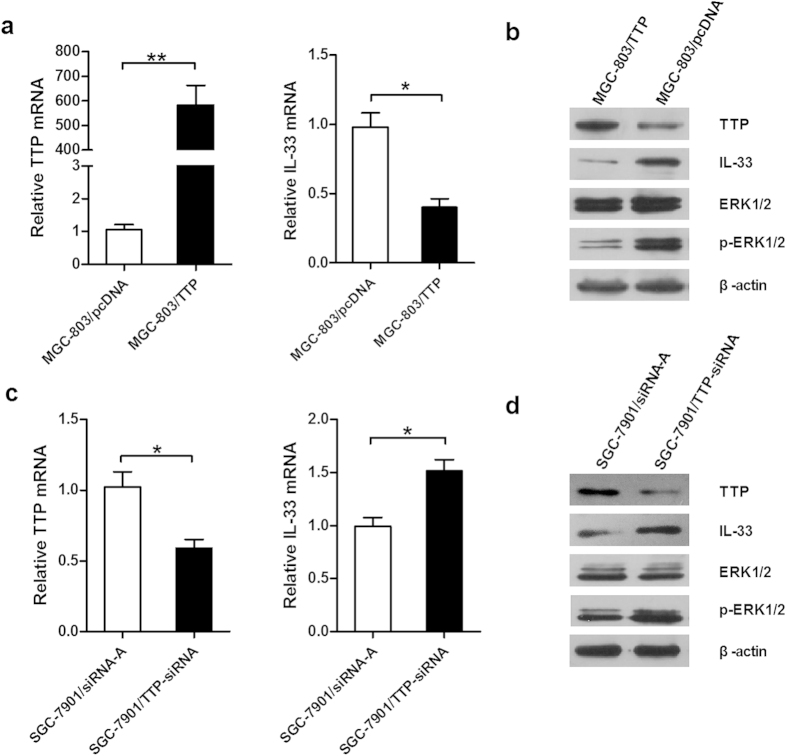
TTP regulates the expression of IL-33 and the activation of ERK1/2 in gastric cancer cells. (**a**,**c**) MGC-803 cells were transfected with pcDNA-TTP or empty vector pcDNA3.1(+); SGC-7901 cells were transfected with TTP-siRNA or control siRNA (siRNA-A). Expressions of TTP and IL-33 in cells were determined by qRT-PCR. (**b**,**d**) Expressions of TTP, IL-33, ERK1/2 and phosphorylation of ERK1/2 (p-ERK1/2) in transfected MGC-803 or SGC-7901 cells were examined by Western blotting. GAPDH and β-actin were used as internal controls for qRT-PCR and Western blotting analysis, respectively. All data are represented as the mean ± SD of three independent experiments. *P < 0.05, **P < 0.01.

As TTP and IL-33 expression levels were inversely correlated in GC, we investigated whether TTP can regulate the level of IL-33. We transfected MGC-803 cells with the TTP expression plasmid pcDNA-TTP to construct a stably-transfected cell line, MGC-803/TTP. As a negative control, MGC-803/pcDNA cells were also generated by transfection with empty vector pcDNA3.1(+). Overexpression of TTP in MGC-803/TTP cells was confirmed by qRT-PCR and Western blotting analysis, and correlated with a significant decrease in IL-33 expression, compared to levels in control cells (Fig. 3a,b).

We further examined whether suppression of TTP would modulate expression of IL-33. As the TTP mRNA level was relatively higher in SGC-7901 than in MGC-803 and BGC-823 cells (Fig. 1c), we transfected SGC-7901 cells with TTP-siRNA to specifically lower the expression of TTP, and the resulting decrease in TTP mRNA and protein levels was associated with enhanced expression of IL-33 (Fig. 3c,d). However, neither the expression of TTP nor of IL-33 was altered after transfecting with non-specific siRNA (siRNA-A).

Since IL-33 has been reported to promote GC cell migration and invasion through activating ERK1/2^12^, we next examined whether ERK1/2 was activated in GC when the expression level of TTP changed. Western blotting analysis showed that up-regulation of TTP in MGC-803/TTP cells decreased the phosphorylation of ERK1/2 (Fig. 3b). Moreover, down-regulation of TTP in SGC-7901 cells by TTP-siRNA increased the phosphorylation of ERK1/2 (Fig. 3d). Overall, these data implied that changes in TTP expression can modulate the expression of IL-33 and the activation of ERK1/2 in GC cell lines.

### TTP inhibits migration and invasion of GC cells *in vitro*

**Figure 4:**
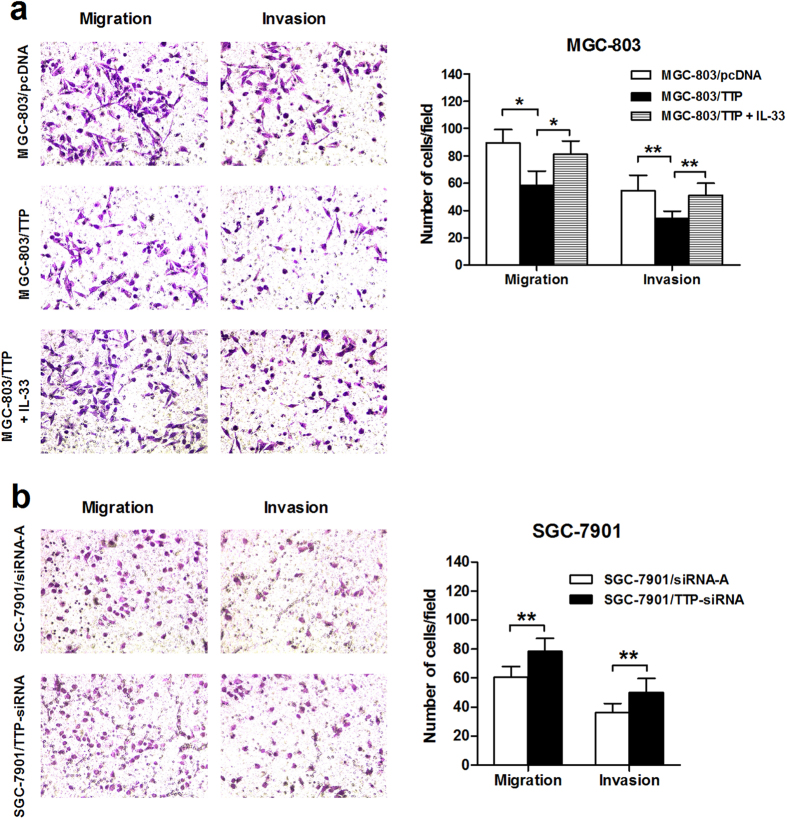
TTP inhibits the migration and invasion of gastric cancer cells *in vitro* through suppression of IL-33 expression. (**a**) Overexpression of TTP inhibited the migration and invasion of MGC-803 cells, while stimulation with recombinant human IL-33 (50 ng/ml) restored them. (**b**) Knockdown of TTP by siRNA enhanced the migratory and invasive abilities of SGC-7901 cells. Pictures shown are representative images of the migration and invasion assays (200× magnification). The data are represented as the mean ± SD of three independent experiments. *P < 0.05, **P < 0.01.

IL-33 is a key player in promoting cell migration and invasion in GC^12^. As an inhibitor of IL-33, the role of TTP in regulating GC metastasis is also of great interest to us as it may have therapeutic potential. The results of transwell assays showed that the migratory (*P* = 0.015) and invasive (*P* = 0.006) abilities of MGC-803/TTP cells were significantly impaired in comparison with MGC-803/pcDNA cells (Fig. 4a).

We also confirmed that down-regulation of TTP expression by TTP-siRNA promoted the migration (*P* = 0.003), and invasion (*P* = 0.002), of SGC-7901 cells (Fig. 4b). Additionally, treatment with recombinant human IL-33 (r-IL-33) abrogated the suppressive effect of TTP on the migration (*P* = 0.038), and invasion (*P* = 0.001), of MGC-803/TTP cells (Fig. 4a). Overall, these data demonstrated that TTP inhibits cell migration and invasion of GC *in vitro*, and that IL-33 is involved in the TTP-mediated suppression of cancer metastasis.

### TTP inhibits proliferation of GC cells *in vitro*

**Figure 5:**
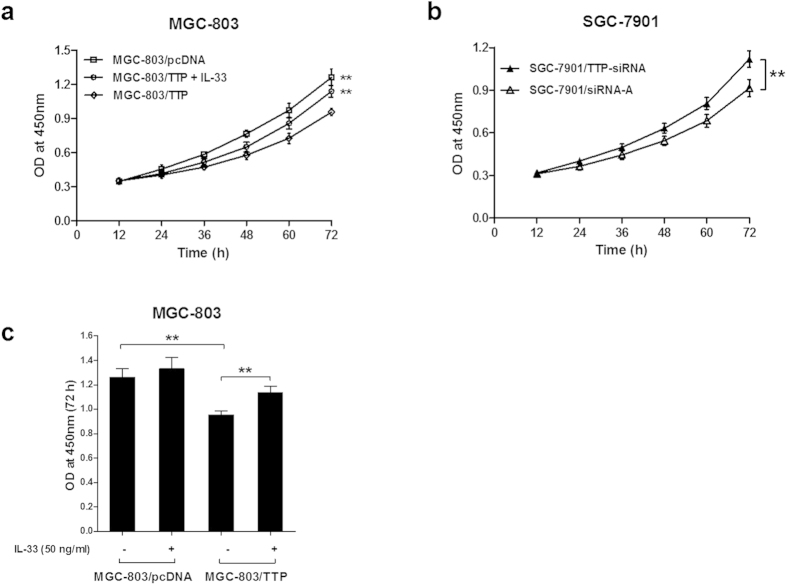
TTP suppresses cell proliferation of gastric cancer *in vitro* through down-regulation of IL-33. (**a**) CCK-8 assay was performed in MGC-803 cells at the indicated times to evaluate the effect of TTP and IL-33 on cell proliferation. (**b**) CCK-8 assay was performed in SGC-7901 cells to evaluate the effect of TTP on cell proliferation. (**c**) Addition of recombinant human IL-33 (50 ng/ml) abolished the inhibitory effect of TTP on the proliferation of MGC-803 cells. The absorbance at 450 nm was measured to assess cell viability. The data are represented as the mean ± SD. **P < 0.01.

Given the tumor-suppressive potential of TTP, we decided to explore the biological functions of TTP in regulating the growth of GC. Using the Cell Counting Kit-8 (CCK-8) assay in MGC-803/TTP and MGC-803/pcDNA cells, we found that overexpression of TTP significantly suppressed the proliferation of GC cells (*P* = 0.002) (Fig. 5a). In contrast, transfection with TTP-siRNA in SGC-7901 cells accelerated the proliferation rate of GC cells, as compared to SGC-7901 cells transfected with siRNA-A (*P* = 0.001) (Fig. 5b).

To test whether TTP-mediated inhibition of cell proliferation correlates with IL-33 levels, MGC-803/TTP and MGC-803/pcDNA cells were treated with r-IL-33 and then analyzed using the CCK-8 assay. The results revealed that the addition of r-IL-33 restored the growth of MGC-803/TTP cells to 90% of the levels of MGC-803/pcDNA cells (*P* = 0.002) (Fig. 5a,c). Although the treatment with r-IL-33 also increased the proliferation of MGC-803/pcDNA cells, the difference was not significant (*P* = 0.334) (Fig. 5c). Taken together, these results indicated that TTP can inhibit the growth of GC *in vitro* through down-regulation of IL-33.

### TTP overexpression suppresses growth of GC *in vivo*

**Figure 6:**
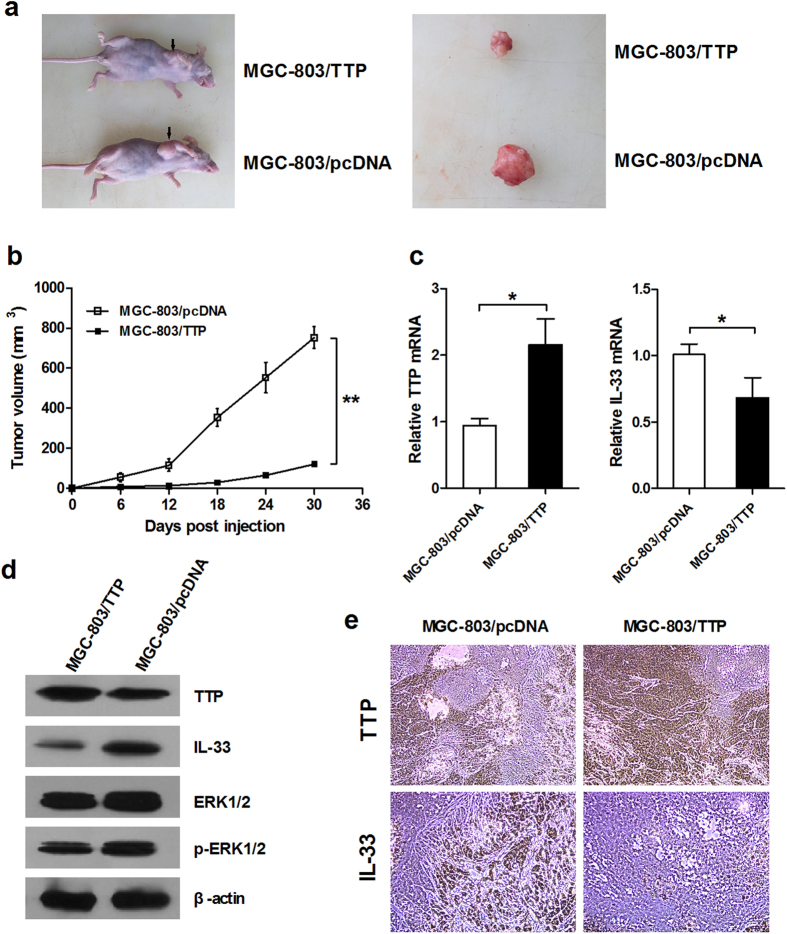
Overexpression of TTP suppresses tumor growth of GC *in vivo*. (**a**) Representative photographs of nude mice (top) and resected tumors (bottom) taken 30 days after injection with MGC-803/TTP or MGC-803/pcDNA cells. (**b**) Volumes of subcutaneous tumors generated by MGC-803/pcDNA or MGC-803/TTP cells. The tumors were measured every 6 days and surgically removed after 30 days of growth. (**c**) qRT-PCR analysis for the expression of TTP and IL-33 in MGC-803/pcDNA and MGC-803/TTP tumor tissues. (**d**) Expressions of TTP, IL-33, ERK1/2 and phosphorylation of ERK1/2 (p-ERK1/2) were examined by Western blotting in subcutaneous tumors of nude mice. (**e**) Immunohistochemical staining (200× magnification) for the expression of TTP and IL-33 in subcutaneous tumors. The data are represented as the mean ± SD. *P < 0.05, **P < 0.01.

We also examined whether overexpression of TTP impacted tumor growth *in vivo*. Equal numbers of MGC-803/TTP or MGC-803/pcDNA cells were injected into the armpits of nude mice and subsequently allowed to grow until tumors formed. By monitoring the volumes of subcutaneous tumors, we discovered that, compared with control cells, cells that overexpressed TTP significantly decreased the growth rate of tumors in nude mice (*P* = 0.003) (Fig. 6a,b). After 30 days of growth, MGC-803/pcDNA cells produced highly vascular tumors, which were markedly larger than those generated by MGC-803/TTP cells (Fig. 6a). Elevated expression of TTP and reduced expression of IL-33 in MGC-803/TTP tumors were confirmed by qRT-PCR, Western blotting analysis and immunohistochemistry (Fig. 6c–e). Additionally, decreased phosphorylation of ERK1/2 was also detected in MGC-803/TTP tumors by Western blotting, suggesting that TTP could inhibit the activation of ERK1/2 *in vivo* (Fig. 6d). The above results suggested that TTP can attenuate growth of GC tumors *in vivo* via suppression of IL-33.

### Correlation of TTP expression with clinicopathological features of GC patients

**Figure 7:**
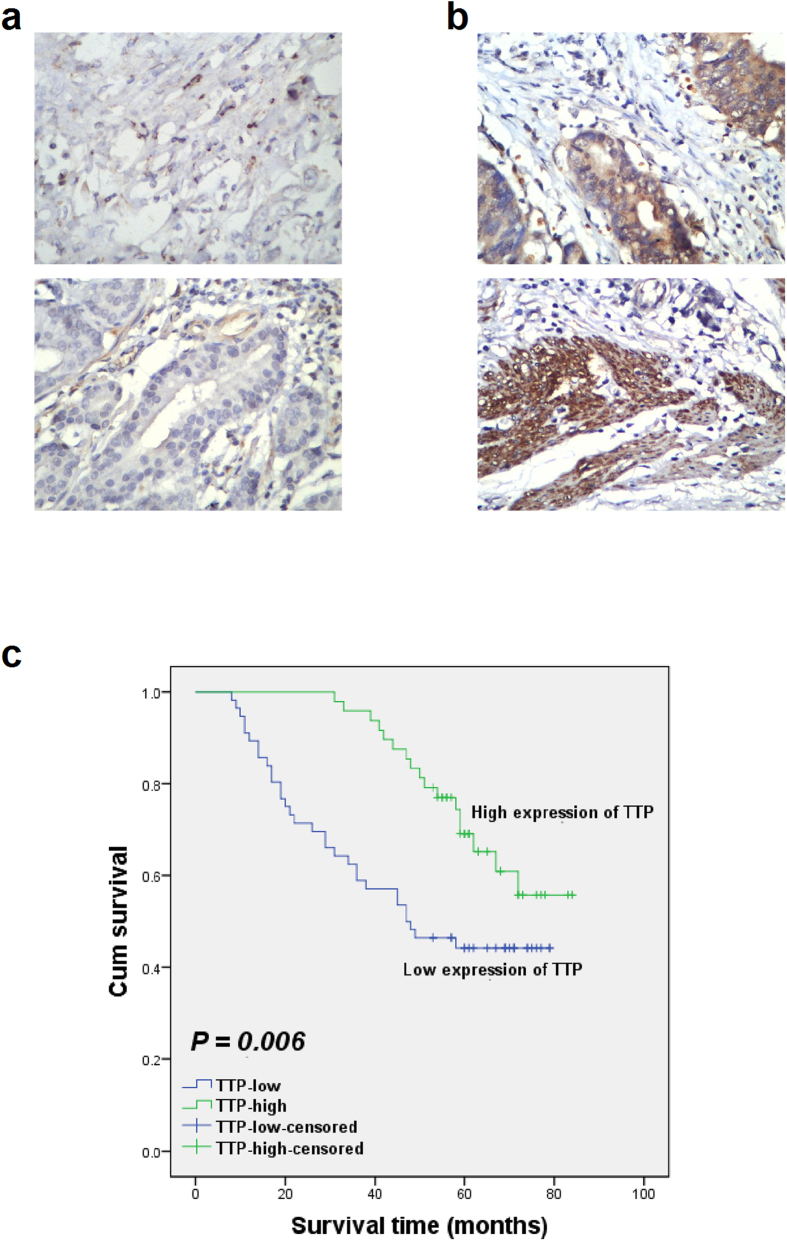
Expression of TTP is correlated with clinicopathological features and overall survival of gastric cancer patients (Group A). (**a**) Representative images of TTP immunohistochemical staining (IHS: top, 1; bottom, 1) for TTP-low subgroup (IHS ≤ 3, n = 56). (**b**) Representative images of TTP immunohistochemical staining (IHS: top, 4; bottom, 6) for TTP-high subgroup (IHS > 3, n = 48). (400× magnification) (**c**) Kaplan-Meier survival curve for gastric cancer patients with different TTP expression levels.

In order to investigate the clinical role of TTP in GC, we analyzed the relationship between TTP expression and clinicopathological factors of the 104 patients from Group A (Table 2). TTP expression levels of all patients were determined by immunohistochemistry (Fig. 7a,b). A median IHS value of 3 was obtained for the patients and was used as the cut-off value to divide the 104 patients into TTP-high (n = 48, IHS > 3) subgroup and TTP-low (n = 56, IHS ≤ 3) subgroup^21^. The clinicopathological features of the patients were summarized by reviewing the pathology reports and clinical histories at the time of surgery. Statistical analysis showed that TTP expression was not significantly correlated with sex (*P* = 0.839), age (*P* = 0.168), differentiation status (*P* = 0.841), or distant metastasis (*P* = 0.066), whereas significant correlations were found between TTP expression and depth of invasion (*P* = 0.001), lymph node metastasis (*P* = 0.010), and the TNM stage (*P* < 0.001) of patients with GC (Table 2).

### Expression level of TTP predicts overall survival of GC patients

Finally, we evaluated the correlation of TTP expression with the overall 5-year survival of GC patients by Kaplan-Meier analysis. A statistically significant correlation was noted between decreased TTP expression and poor survival, with a mean survival time of 60.19 months (95% CI: 56.55–63.83; median survival time: 60.00 months) for 48 patients with relatively high TTP expression, as compared to 44.82 months (95% CI: 38.57–51.07; median survival time: 47.50 months) for 56 patients with relatively low TTP expression (Fig. 7c, log-rank test: *P* = 0.006). Information of post-operation therapy was also obtained: 12 patients from Group A did not receive any post-operation therapy (mean survival time: 64.00 months, 95% CI: 54.34–73.66; median survival time: 70.50 months), while the rest 92 patients received post-operation chemotherapy (mean survival time: 50.34 months, 95% CI: 46.06–54.62; median survival time: 56.00 months). Statistical analysis showed that there was no significant correlation between post-operation therapy and the survival of GC patients (*P* = 0.106).

In addition, univariate Cox’s regression analysis indicated that the differentiation status (*P* = 0.004), depth of invasion (*P* = 0.019), lymph node metastasis (*P* < 0.001), distant metastasis (*P* < 0.001), TNM stage (*P* < 0.001), and TTP expression (*P* = 0.008) could be used to predict the overall survival of GC patients, with results indicating that patients with poor differentiation, deep invasion, lymph node metastasis, distant metastasis, advanced TNM stage, or reduced TTP expression had poor survival rate (Table 3). Moreover, multivariate Cox’s proportional hazards regression analysis further identified differentiation status (HR = 2.756, *P* = 0.007), distant metastasis (HR = 6.624, *P* = 0.001) as well as TTP expression (HR = 2.152, *P* = 0.029) to be three independent prognostic factors in our study (Table 3). All of the above analyses implicated that reduced TTP expression level was associated with poor prognosis of patients with GC.

## Discussion

It has been well established that the microenvironment has a profound influence on cellular tumorigenesis. Inflammatory and immune cells in tumors, such as dendritic cells, macrophages, and lymphocytes can induce disruption of the microenvironment by producing tumor-promoting cytokines and other factors. An representative example is associated with human gastric cancer, where inflammation caused by the bacterium *Helicobacter pylori* remains a major risk factor for cancer development^22^,^23^. Dysregulation of multiple inflammatory cytokines, such as IL-6, IL-8, IL-10, IL-16, IL-17, and IL-33, has also been shown to be closely associated with the initiation and development of GC^4^,^11^,^24^,^25^,^26^.

The pro-inflammatory cytokine IL-33 exerts its biological function by binding to the ST2 receptor, thus activating multiple signaling proteins, such as NF-κB, inhibitor of NF-κB-α, ERK1/2, MAPK and JNK1/2, resulting in the induction of the inflammatory mediators IL-1β, IL-3, IL-6, tumor necrosis factor (TNF), IL-4, IL-5, and IL-13^5^,^6^,^7^,^27^,^28^. Ectopic expression of IL-33 has been reported in hepatocellular carcinoma, and is related to tumor growth and metastasis^8^. In breast cancer, the IL-33/ST2 pathway has been found to accelerate cancer progression via enhanced intratumoral accumulation of immunosuppressive cells, and by the suppression of innate antitumor immunity^9^. Furthermore, the IL-33/ST2 axis also enhanced the phosphorylation of mitogen-activated protein kinase kinase kinase 8 (MAP3K8), resulting in the promotion of epithelial cell transformation and breast tumorigenesis^29^. Additionally, expression of IL-33 is increased in the sera and tumor tissues of GC patients, and this increase correlates with depth of invasion, distant metastasis, advanced tumor stage, and poor survival^11^. Further investigations revealed that IL-33 induces the activation of the ERK1/2 pathway via the ST2 receptor, subsequently increasing the secretion of MMP-3 and IL-6 to promote the invasion and migration of GC cells^12^. Consistent with the above findings, our immunohistochemistry results also demonstrate that IL-33 expression is relatively higher in gastric cancer tissues than in adjacent normal tissues, further supporting the tumor-promoting effect of IL-33 in GC.

The inflammatory response requires coordinated regulation of pro-inflammatory proteins. Transcriptional activation is essential for the synthesis of mRNA that encodes inflammatory proteins, while the level of protein expression is determined by posttranscriptional regulation of mRNA stability and translation. Commonly, these mechanisms jointly dampen inflammation by suppressing the overexpression of potentially injurious pro-inflammatory proteins. Most pro-inflammatory transcripts that are regulated at the posttranscriptional level possess AREs, within their 3′UTR, that are capable of inducing mRNA destabilization through specific binding to ARE-binding proteins^14^. TTP is a well-characterized ARE-binding protein, which promotes ARE-mRNA decay via multiple pathways, including enhanced deadenylation of ARE-mRNA as well as further interactions with the exosome, the decapping/Xrn1 complex, and the RNA-induced silencing complex^14^,^30^.

It has been reported that TTP can bind to the 3′UTR of IL-23 mRNA and promote degradation of IL-23 mRNA, leading to the suppression of IL-23 expression in colon cancer cells^16^. In addition to acting as a posttranscriptional regulator by affecting mRNA stability, TTP can also control the expression of inflammatory mediators at the transcriptional level. Independent of its mRNA-destabilizing ability, TTP inhibits the transcriptional activity of NF-κB by blocking nuclear translocation of the p65 subunit, and by recruiting histone deacetylases to the promoters of NF-κB target genes. By suppressing NF-κB signaling, TTP further down-regulates the expression of NF-κB-dependent genes, such as *IL-1β*, *IL-12*, and *TNF-α*^31^,^32^,^33^. Although we did not directly examine the mechanisms underlying the regulation of IL-33 by TTP in this study, our observations indicated the inhibitory effect of TTP on IL-33 expression by providing evidence that ectopic expression of TTP in GC markedly reduced IL-33 expression, whereas the suppression of TTP by siRNA increased IL-33 levels. Consistent with the report that IL-33 could induce the activation of ERK1/2 in GC, we found that up-regulation of TTP decreased the phosphorylation of ERK1/2, implicating that TTP could suppress the expression of IL-33 and the activation of ERK1/2 at the same time.

Because TTP is mainly expressed in activated macrophages and T-cells, the importance of TTP in the regulation of human diseases was originally established in connection to the immune system^14^. However, recent studies have also identified TTP as a tumor suppressor that limits the expression of various tumor-promoting regulators. Reduced expression or inactivation of TTP has been found in different types of cancers, and is associated with the poor prognosis of cancer patients^19^,^20^,^34^. Our study provides the first report on the inhibitory effect of TTP on GC development. We demonstrated that TTP expression is aberrantly down-regulated in GC tissues and cells, and that enhanced expression of TTP significantly suppressed the proliferation, migration, and invasion of GC cells *in vitro*. Experiments with nude mice further confirmed that TTP inhibits tumor growth *in vivo*. Moreover, treatment with recombinant human IL-33 abrogated the TTP-mediated inhibition of tumor growth and metastasis, suggesting that the tumor-suppressive role of TTP in GC is associated with IL-33 suppression.

Although considerable efforts have been made to improve the diagnosis and treatment of GC, the prognosis of patients with advanced GC is still poor. Malignant proliferation, extensive invasion, and lymphatic metastasis have long been major challenges that limit effective therapeutic strategies. Therefore, identifying potential markers for the early detection of GC, and for determining the prognosis of GC patients, is critical for improving outcomes. Most of the GC prognostic biomarkers in clinical practice are serum-based, such as carcinoembryonic antigen (CEA), carbohydrate antigen 19-9 (CA19-9), and carbohydrate antigen 72-4 (CA72-4)^35^,^36^. Gastric cancer associated antigen (MG7-Ag) is another marker in both serum and tissues from GC patients, which has been reported to have relative higher sensitivity and specificity for detecting GC^37^. Besides, transcription factors, receptors, enzymes, and cytokines are also important members of the prognostic biomarkers^35^. For example, c-Myc is a transcription factor that is correlated with metastasis and poor prognosis in GC^38^. Vascular endothelial growth factor (VEGF) is also a tumor marker that is overexpressed in many cancers including GC, it is an angiogenic factor which promotes endothelial cell proliferation and migration and induces endothelial cell angiogenesis^39^,^40^. Recent studies have also identified microRNAs (miRNAs) and long non-coding RNAs (lncRNAs) as novel candidates for GC biomarkers, such as *miR-122*, *miR-21*, *miR-148a*, lncRNA *H19*, *HOTAIR*, *CCAT1*, etc^41^,^42^,^43^,^44^. Similar to miRNAs, RNA binding proteins can also regulate the mRNA stability, splicing, translation and transport at post-transcriptional level^45^. Therefore, the potential of RNA binding proteins as tumor markers has attracted great interest recently. For instance, RNA binding protein Quaking (QKI) has been found to be significantly down-regulated in GC. The expression of QKI is associated with the clinicopathologic characteristics and the prognosis of GC, which serves as an independent prognostic factor for survival of GC patients^46^. Our results showed that the expression of RNA binding protein TTP is closely correlated with depth of invasion, lymph node metastasis, and TNM stage. Kaplan–Meier analysis revealed a positive relationship between TTP expression and overall survival of GC patients, whereas lower TTP expression indicated shorter survival time. Furthermore, multivariate Cox’s proportional hazards model suggested that reduced TTP expression was an independent prognosis predictor for poor survival. Overall, these analyses implied that decreased levels of TTP predicted a poor prognosis for GC patients.

In summary, our study reveals that lower expression of TTP contributes to GC progression, while elevated expression of TTP inhibits the proliferation, migration, and invasion of GC cells through suppression of IL-33. Additionally, reduced TTP expression is associated with depth of invasion, lymph node metastasis, advanced TNM stage, and poor survival. Our findings demonstrate the potential of TTP as a therapeutic target for GC treatment, and as a diagnostic and prognostic indicator for GC patients. However, further experiments are still needed to explore the underlying mechanism of TTP in the regulation of IL-33. Besides, studies with larger sample size and more specific post-operation therapy information will also help to offer better understandings of the diagnostic and prognostic values of TTP in GC.

## Materials and Methods

### Patients and tissue samples

This study was approved by the Ethics Committee of Nanjing Medical University and informed consent was obtained from all patients involved in the study. All the experimental methods in the current study were carried out in accordance with the approved guidelines by the Ethics Committee of Nanjing Medical University.

Paraffin-embedded tumor specimens from 104 patients (Group A) who underwent resection of gastric carcinoma between July 2007 and February 2010 were collected for immunohistochemistry. Fresh tumor tissues and adjacent normal tissues, from 70 patients (Group B) who underwent surgical treatment for GC from October 2012 to January 2015, were also obtained for RNA and protein extraction, or immunohistochemistry. The fresh tissue samples were immediately immersed in liquid nitrogen for 5 min after surgical resection and then stored at −80 °C until further processing. All participants were randomly selected from the patients diagnosed with GC, at Nanjing Medical University Affiliated Wuxi Second Hospital, who had not received chemotherapy or radiation therapy prior to surgery. Tumor stages of participants were determined according to the Cancer Staging Manual (Seventh Edition) of the American Joint Committee on Cancer (AJCC).

### Cell culture

Three human GC cell lines (SGC-7901, BGC-823, and MGC-803) were purchased from Cell Resource Center, Institute of Basic Medical Sciences, Chinese Academy of Medical Sciences. The human gastric epithelial cell line GES-1 was preserved in our laboratory. MGC-803 was cultured in Dulbecco’s modified eagle medium (DMEM) (Gibco-BRL, USA) supplemented with 10% fetal bovine serum (FBS) (HyClone, USA), while others were maintained in RPMI-1640 (Gibco-BRL, USA) with 10% FBS. All cells were cultured at 37 °C in a humidified incubator with 5% CO_2_. Culture medium was changed three times a week.

### RNA extraction and qRT-PCR

Total RNA was extracted and purified from cells and tissue samples using TRIzol Reagent (Invitrogen, USA), according to the manufacturer’s instructions. The cDNA was then synthesized using the PrimeScript RT Reagent Kit with gDNA Eraser (Takara, Dalian, China). We performed qRT-PCR using the QuantiFast SYBR Green PCR Kit (Qiagen, Germany) on an ABI StepOnePlus Fast real-time PCR system (Applied Biosystems) to assess mRNA expression. The specificities of amplification products were confirmed by melting curve analysis and agarose gel electrophoresis, and GAPDH was used to normalize the mRNA levels. PCR primers were purchased from Invitrogen (Shanghai, China), and the sequences were as follows: TTP: CGCTACAAGACTGAGCTAT, GAGGTAGAACTTGTGACAGA; IL-33: GACTCCTCCGAACACAGAGC, CCCAGCTTGAAACACAAGGC; GAPDH: ACGGATTTGGTCGTATTGGGC, TTGACGGTGCCATGGAATTTG. The relative expression levels of the target genes were quantified by the 2^−ΔΔCT^ method, and all samples were measured in triplicate.

### Plasmid construction, siRNA, and cell transfection

Full-length human cDNA of TTP was synthesized and integrated by Invitrogen (Shanghai, China), and the product was then subcloned into the *Hin*dIII and *Eco*RI sites of the pcDNA3.1(+) vector (Invitrogen) to construct plasmid pcDNA-TTP. After confirmation by sequencing by Invitrogen (Shanghai, China), pcDNA-TTP was transfected into MGC-803 using Lipofectamine 2000 (Invitrogen, Shanghai, China) to generate MGC-803/TTP cells. Stably-transfected cells were selected by adding G418 (350 μg/ml; Invitrogen) two days after transfection. After 14 days of screening, stable transfectants were selected for further amplification, and were then tested by qRT-PCR or Western blotting for overexpression of human TTP. The control cell line MGC-803/pcDNA was also generated by transfection with the empty pcDNA3.1(+) vector.

Using Lipofectamine 2000, 100 nM of either TTP-siRNA (sc36761, Santa Cruz Biotechnology) or control siRNA-A (sc37007, Santa Cruz Biotechnology) was transfected into SGC-7901 cells cultured in 6-well plates at 3 × 10^5^ cells/ml. At 48 h post-transfection, cells were harvested for either qRT-PCR or Western blotting to analyze the knockdown efficiency.

### Protein extraction, antibodies, and Western blotting

Cells or tissue samples were lysed in radioimmunoprecipitation assay (RIPA) lysis buffer (Proteintech, USA) with phenylmethylsulfonyl fluoride (PMSF) (1 mM, final concentration). Extracted proteins were separated by SDS-PAGE and transferred to PVDF membranes, which were then incubated overnight at 4 °C in appropriate dilutions of anti-TTP rabbit polyclonal antibody (ab33058, Abcam) or anti-IL-33 mouse monoclonal antibody (ab54385, Abcam), followed by, respectively, goat anti-rabbit IgG or goat anti-mouse IgG (Proteintech, USA), as secondary antibodies. Immobilon^TM^ Western Chemiluminescent HRP Substrate (Millipore, USA) was used for detection, and anti-β-actin antibody (Proteintech, USA) was used as an internal control.

### Exposure to recombinant human IL-33

Recombinant human IL-33 (r-IL-33) was obtained from ACRObiosystems (Newark, DE, USA). Cells were incubated with 50 ng/ml of recombinant human IL-33 and used for additional experiments at the indicated times.

### Cell proliferation assay

For cell proliferation assays, approximately 3 × 10^3^ transfected cells were seeded in 96-well plates. Cell Counting Kit-8 (CCK-8) reagent (Dojindo Laboratories, Kumamoto, Japan) was then added to each well in accordance with the manufacturer’s instructions. The absorbance of each well at 450 nm was measured by Multiskan GO (Thermo Fisher Scientific) to calculate cell counts after 12 h, 24 h, 36 h, 48 h, 60 h, and 72 h of culture. This assay was replicated three times.

### Cell migration and invasion assay

Transwell inserts with 8.0 μm pore polycarbonate membranes (Corning Costar, USA) were used for the migration assay and Transwell inserts coated with Matrigel (BD Biosciences, USA) were used for the invasion assay. Approximately 2 × 10^5^ transfected cells were seeded into the upper chamber of an insert and cultured in medium with 1% FBS, while medium with 10% FBS was added into the bottom chamber as a chemoattractant. After several hours of incubation, cells on the upper surface of the membrane were removed with a cotton swab. Next, cells on the lower surface of the membrane were fixed by methanol and stained with crystal violet (Sigma, USA). The average number of stained cells was counted from five different fields of each well using an optical microscope (200× magnification). Both assays were repeated three times.

### Immunohistochemical staining assay

For immunohistochemistry, 4 μm-thick sections of the paraffin-embedded specimens were placed into xylene and then into a graded series of ethanol for deparaffinization and rehydration. The sections were then heated with sodium citrate buffer and boiled at 100 °C for 2 min for antigen retrieval. After being washed in phosphate buffered saline with Tween-20 (PBST), the sections were incubated overnight at 4 °C with 1:100 diluted anti-TTP rabbit polyclonal antibody or anti-IL-33 mouse monoclonal antibody. Primary antibodies were detected using MaxVision HRP-Polymer anti-Mouse/Rabbit IHC Kit (Maixin Biotech, China). DAB kit (Maixin Biotech, China) was applied for color development, and the sections were subsequently counterstained with hematoxylin. For negative controls, the primary antibodies were omitted.

To evaluate the expression of TTP and IL-33, a semiquantitative method based on both staining intensity and proportion of staining was utilized. Staining intensity was scored as: 0, negative; 1, weak; 2, moderate; 3, strong. The proportion of staining was scored as: 1, 0–25%; 2, 26–50%; 3, 51–75%; 4, 76–100%. The final immunohistochemistry score (IHS) was obtained by multiplying the staining intensity by the proportion of staining^20^. Brown cytoplasmic staining was considered to indicate positive immunoreactivity. Two pathologists, who were blinded to clinicopathological information on patients, independently evaluated the IHS of each section under optical microscopes (100×, 200×, and 400× magnification), assessing five different fields of each section to calculate the average IHS.

### Survival analysis

The 104 patients from Group A were enrolled in the survival analysis. Follow-up surveys were made by telephone, visits, or letters to update the information and survival data of the patients. The survey period was completed by April 2015, with five years of follow-up records obtained for each patient. Overall survival time was defined as the time from the date of surgery to death (for non-censored events) or to the end of the survey period (for censored events).

### Generation and analysis of tumors

A total of 20 six-week-old (BALB/c-nu) nude mice (Cavens Laboratory, Changzhou, China) were randomly divided into two groups of 10 mice each. Cell suspensions of MGC-803/TTP or MGC-803/pcDNA with equal concentrations (1 × 10^7^ cells/100 μl) were then injected subcutaneously into the mice. The generated tumors were measured using calipers every six days, and tumor volumes were calculated with the formula: volume = (length × width^2^)/2^47^. After monitoring the tumor growth for 30 days, the nude mice were sacrificed and the tumor tissues were removed surgically, and were then either frozen for RNA extraction or fixed in 10% formalin for immunohistochemistry. All experiments with mice were approved by the Animal Care and Use Committee of Nanjing Medical University.

### Statistical analysis

All statistical analyses were performed using SPSS 19.0 (SPSS, USA). Correlations between TTP expression and clinical variables were assessed by Pearson’s χ^2^ test or Fisher’s exact test. Other data were evaluated by Student’s t-test or one-way ANOVA. The survival curve was estimated by the Kaplan-Meier method, along with the log-rank test to assess statistical significance^21^. Cox’s proportional hazards regression model was performed to calculate hazard ratios (HR) and their 95% confidence intervals (CI)^21^. A *P* value of less than 0.05 was considered statistically significant and all *P* values were two-sided. All data were represented as the mean ± SD.

## Additional Information

**How to cite this article**: Deng, K. *et al.* Tristetraprolin inhibits gastric cancer progression through suppression of IL-33. *Sci. Rep.* **6**, 24505; doi: 10.1038/srep24505 (2016).

## Figures and Tables

**Table 1:** Correlation between TTP expression and IL-33 expression in GC tissues (Group B).

Variable	N	TTP expression				χ^2^	*P* Value
		TTP-positive	%	TTP-negative	%		
IL-33 expression						17.188	**<0.001**
IL-33-positive	50	11	22.0	39	78.0		
IL-33-negative	20	15	75.0	5	25.0		
N		26		44			

*P* value < 0.05 is indicated in bold.

**Table 2:** Correlation between TTP expression and clinicopathological features of GC patients (Group A).

Variable	N	TTP expression				*P* Value
		TTP-low	%	TTP-high	%	
Sex						0.839
Male	65	34	52.3	31	47.7	
Female	39	22	56.4	17	43.6	
Age						0.168
>60	54	33	61.1	21	38.9	
≤60	50	23	46.0	27	54.0	
Differentiation status						0.841
Well/Moderate	66	35	53.0	31	47.0	
Poor/Undifferentiated	38	21	55.3	17	44.7	
Depth of invasion						**0.001**
T1–T3	53	20	37.7	33	62.3	
T4	51	36	70.6	15	29.4	
Lymph node metastasis						**0.010**
Absent (N0)	48	19	39.6	29	60.4	
Present (N1 + N2 + N3)	56	37	66.1	19	33.9	
Distant metastasis						0.066^a^
Absent (M0)	96	49	51.0	47	49.0	
Present (M1)	8	7	87.5	1	12.5	
TNM stage						**<0.001**
I + II	55	18	32.7	37	67.3	
III + IV	49	38	77.6	11	22.4	

^a^*P* value when expression levels were compared using Fisher’s exact test.

*P* values < 0.05 are indicated in bold.

**Table 3:** Univariate and multivariate analyses for overall survival of GC patients (Group A).

Variable	Unadjusted HR^a^ (95% CI)	*P* Value	Adjusted HR^b^ (95% CI)	*P* Value
Sex	0.719 (0.404–1.278)	0.261	1.052 (0.563–1.965)	0.874
Age	0.883 (0.501–1.559)	0.668	1.671 (0.797–3.503)	0.174
Differentiation status	2.330 (1.311–4.143)	**0.004**	2.756 (1.328–5.721)	**0.007**
Depth of invasion	1.991 (1.119–3.540)	**0.019**	0.627 (0.265–1.483)	0.287
Lymph node metastasis	3.302 (1.742–6.257)	**<0.001**	2.316 (0.822–6.530)	0.112
Distant metastasis	14.535 (6.117–34.540)	**<0.001**	6.624 (2.236–19.621)	**0.001**
TNM stage	3.071 (1.695–5.564)	**<0.001**	1.648 (0.510–5.328)	0.404
Post-operation therapy	0.395 (0.122–1.274)	0.120	0.665 (0.171–2.582)	0.555
TTP expression	2.237 (1.236–4.051)	**0.008**	2.152 (1.081–4.283)	**0.029**

^a^Hazard ratios in univariate models.

^b^Hazard ratios in multivariable models.

*P* value < 0.05 was indicated in bold.
